# Rongbei Maimendong Decoction Promotes Radiosensitivity of Non‐Small Cell Lung Cancer Cells by Inhibiting SOD1 Expression

**DOI:** 10.1155/mi/3930562

**Published:** 2025-12-08

**Authors:** Shilong Liu, Xueying Pang, Liqun Wang, Ning Zhan, Shangjie Wu, Jiaxing Deng, Ke Jin, Yang Bai, Deyou Jiang, Lishuang Qi, Zhuying Li

**Affiliations:** ^1^ Department of Respiratory, First Affiliated Hospital, Heilongjiang University of Chinese Medicine, Harbin, China, hljucm.edu.cn; ^2^ Department of Thoracic Radiation Oncology, Harbin Medical University Cancer Hospital, Harbin, China, hrbmu.edu.cn; ^3^ Department of Oncology, First Affiliated Hospital, Heilongjiang University of Chinese Medicine, Harbin, China, hljucm.edu.cn; ^4^ Department of Radiation Oncology, Xiang’an Hospital of Xiamen University, Xiamen, China, xmu.edu.cn; ^5^ College of Bioinformatics Science and Technology, Harbin Medical University, Harbin, China, hrbmu.edu.cn; ^6^ College of Basic Medical Science, Heilongjiang University of Chinese Medicine, Harbin, China, hljucm.edu.cn

**Keywords:** β-thymidine, NSCLC, radiosensitivity, Rongbei Maimendong decoction (RBMD), SOD1

## Abstract

Some natural remedies in traditional Chinese medicine (TCM) inhibit tumor progression and increase sensitivity in the treatment of patients who have received radiation therapy. However, the specific synergistic effect of the active ingredients in the traditional Chinese therapeutic Rongbei Maimendong decoction (RBMD) on sensitivity to radiation therapy for non‐cell lung cancer (NSCLC) is still unclear. RBMD inhibited transplanted tumor cell growth in mice, showing a dose‐dependent effect in tumor growth inhibition following radiotherapy. Our study utilized bioinformatics analysis and liquid chromatography with tandem mass spectrometry to identify the presence of β‐thymidine (β‐thy) in *Phellodendron* bark, the primary component of RBMD. This compound modulated mRNA and protein levels of SOD1 by interacting with SOD1, inhibiting the malignant characteristics (proliferation, apoptosis, migration, and invasion) of NSCLC cells. Furthermore, apoptosis assays demonstrated that β‐thy attenuated tumor growth in mice following radiotherapy. Single‐cell gel electrophoresis assays demonstrated the ability of SOD1 to mitigate DNA damage in irradiated (IR) NSCLC cells, suppressing apoptosis, and that β‐thy attenuated this SOD1 protective effect, mitigating NSCLC cells resistance to radiation. We propose that the consumption of RBMD, which is rich in β‐thy, may enhance NSCLC radiosensitivity by hindering DNA repair mechanisms and facilitating apoptosis through DNA damage augmentation.

## 1. Introduction

Lung cancer has the highest incidence and mortality worldwide, with a mere 5‐year survival rate of 26% for patients with non‐cell lung cancer (NSCLC) [1]. This low survival rate may reduce the radiotherapy response due to the tendency of patients to be diagnosed in the progressive stages [2]. Furthermore, immunotherapy utilization remains limited, underscoring the importance of investigating the molecular mechanisms underlying resistance to radiotherapy and chemotherapy in NSCLC and identifying potential candidates for immunotherapy. This study is critical to improving clinical treatment strategies and extending survival in NSCLC patients.

Our previous experiments [3, 4] demonstrated that CBR3‐AS1 regulates reactive oxygen species (ROS) levels and DNA damage repair by regulating the expression of miR‐409‐3p‐mediated SOD1 and affects radiosensitivity in NSCLC cells. CBR3‐AS1 binds miR‐409‐3p to regulate the expression of its target gene. The epigenetic factor SETDB1 is instrumental in modulating the influence of the miR‐409‐3p/SOD1 pathway on NSCLC radiosensitivity. However, additional research is warranted to assess the potential effects of traditional Chinese medicine (TCM) treatments on NSCLC radiation sensitivity.

Various TCM remedies stimulate the body’s immune response and exhibit antitumor effects [5]. For example, curcumin specifically inhibits the NF‐κB‐mediated DNA repair pathway, induces G2/M phase arrest (the radiation‐sensitive phase of cell division), and increases radiation‐induced DNA damage via PARP cleavage upregulation, showing precise targeting with clear mechanisms [3, 4]. Resveratrol displays unique dual‐phase regulation‐activating NAD+‐dependent deacetylase SIRT1 to control DNA repair and metabolism while blocking tumor escape pathways like STAT3 and PI3K/Akt. The dose‐dependent ability to control oxidative stress provides a unique mechanism of radiosensitization [6, 7]. Patients after radiotherapy and chemotherapy for lung cancer often show a condition classified in Chinese medicine as “lung flaccidity” due to gas stagnation and pulmonary disorders. Additionally, after radiotherapy, patients with chest tumors frequently experience secondary symptoms, such as coughing, asthma, throat discomfort, excessive phlegm or saliva, red tongue, and reduced tongue fur, together with a speed‐deficient pulse, indicating that “Yin deficiency with internal Heat” is an important etiological and mechanical characteristic of chest radiation injury. A primary clinical manifestation in patients with radiation‐induced lung injury is Lung‐Stomach Yin deficiency, accompanied by an upward‐flaming deficiency Fire. The text *Synopsis of Prescriptions of The Golden Chamber on the original meaning* describes this as a condition characterized by the counterflow of fire and ascending gas, resulting in heat and gas rushing upwards, causing throat discomfort due to lung dryness, impairing fluid production. *Ophiopogonis* is primarily administered to promote fluid production and alleviate dryness, with *Pinelliae* serving as an adjunct to alleviate obstruction. Additionally, ginseng, *Glycyrrhizae*, *Semen Oryzae Nonglutinosae*, and Fructus *Ziziphi Jujubae* are prescribed to tonify and nourish the stomach, strengthen the Earth element, and promote the Metal element (related to the stomach and lung in TCM theory). This treatment approach is effective for addressing lung deficiency accompanied by pathogenic heat and depletion of body fluids as well as for early intervention in cases of pulmonary weakness with lung deficiency and pathogenic heat. Decoction of *Radix Ophiopogonis* improves the side effects of radiotherapy in patients with malignant tumors, and shows that the curative effect is significant [8].

The decoction used in this study consisted primarily of *R. Ophiopogonis*, a traditional prescription found in the *Synopsis of Prescriptions of The Golden Chamber*. Additionally, it contains medicinal plants typical of the black soil region around Longjiang Medical School [9], such as Jilin matsutake [10], northeastern fritillary [11], and northern *Taxus chinensis* [12]. The final concoction exhibits high quality and unique medicinal attributes. Therefore, the unique cancer diagnosis method and anticancer treatment approach in Longjiang are associated with the decoction used in this study. Moreover, with the renewal of the concept of health preservation in TCM, people have a deeper understanding of the theory relating the homology of medicine and food. As expressed in *The Yellow Emperor’s Classic of Internal Medicine*: “Grains for nourishing, fruits for assisting, livestocks for benefiting, vegetables for filling, and a harmonious combination of gas and flavor are for tonifying essence and replenishing gas.”

Herein, japonica rice, which is often used in the decoction of *R. Ophiopogonis*, was replaced by Longjiang characteristic rice, adhering to the concept of homology of medicine and food for tumor prevention and treatment and in accordance with the concepts of TCM. The theory of homology of medicine and food has been widely used in clinical and basic research to strengthen anticancer therapy and enhance immunity after radiotherapy and chemotherapy in NSCLC.

In combination with chemotherapy, *Taxus chinensis*, which is frequently used in TCM as the active component of Rongbei Maimendong decoction (RBMD), inhibits NSCLC cells proliferation, promotes apoptosis, and reverses drug resistance through multiple pathways [12]. However, the synergistic effect between the active components of RBMD on NSCLC cell radiosensitivity remains unclear.

Tumor cells interact with the microenvironment through crosstalk. Tumor‐related immune response is an important factor influencing this tumor microenvironment. TCM enhances the effectiveness of anti‐cancer treatments and strengthens the patient’s immune system. Moreover, TCM is an important bridge in the conversation between tumor‐related immune responses and the exchange of information via the RNA pathway [13]. Therefore, TCM is an important component of tumor adjuvant treatment. Basic and clinical studies have shown that TCM treatments inhibit tumor progression and improve sensitivity to radiation therapy, chemotherapy, and targeted therapies. This study aimed to analyze the mechanisms of regulation of radiosensitivity in NSCLC cells by RBMD active ingredients.

## 2. Materials and Methods

### 2.1. Cell and Animal Culture

In this investigation, several human cell lines were utilized: H460, PC9, H520, A549, H1299, H1650, and BEAS‐2B human lung epithelial cells. All cell lines were purchased from Sabancon Biotechnology Corporation (Shanghai, China). BEAS‐2B cells were cultured in Dulbecco’s modified Eagle medium (DMEM) containing 10% fetal bovine serum (FBS), whereas the remaining cell lines were cultured in RPMI‐1640 medium containing 10% FBS. All cells were incubated at 37 °C with 5% CO_2_.

For the in vivo component of the study, a total of 15 male C57BL/6 mice were sourced from Liaoning Changsheng Biotechnology Co. Ltd. These mice were in a controlled environment that followed a 12‐h light and 12‐h dark cycle, with a temperature maintained at 22 ± 1 °C, and a humidity at 45%–55%. They were provided with autoclaved standard chow and sterile water ad libitum.

### 2.2. Bioinformatics Analyses

#### 2.2.1. Rongbei Maimendong‐Related Resources

The information about the herbs and compounds that constitute Rongbei Maimendong was obtained from the Encyclopedia of TCM (ETCM) database (http://www.tcmip.cn/ETCM/) [14], such ingredients include renshen, dazao, banxia, gancao, maidong, and pingbeimu.

#### 2.2.2. NSCLC‐Related Resources

RNA sequencing (RNA‐seq) data of patients with lung adenocarcinoma (LUAD) and lung squamous cell carcinoma (LUSC) were obtained from The Cancer Genome Atlas (TCGA; last accessed May 1, 2022, https://portal.gdc.cancer.gov/) [15]. TCGA‐LUAD data included 539 LUAD and 59 normal samples, and TCGA‐LUSC data included 502 LUSC and 51 normal samples. The fragments per kilobase of transcript per million mapped read values for RNA‐seq profiles were log2‐scaled + 1 for gene expression measurements [16]. The independent datasets (GSE19804 and GSE32863) for NSCLC samples and paired adjacent non‐tumor lung tissues (normal controls) were downloaded from the Gene Expression Omnibus (GEO, http://www.ncbi.nlm.nih.gov/geo/). GSE19804 includes 60 NSCLC samples and paired normal controls, and GSE32863 includes 58 NSCLC samples and paired normal controls.

#### 2.2.3. Bioinformatics Methods

Student’s *t*‐test was used to identify differentially expressed genes (DEGs) between two groups. Functional enrichment analysis was performed using a cumulative hypergeometric distribution model based on functional pathways in the Kyoto Encyclopedia of Genes and Genomes (KEGG). The genes related to immunity [17], stemness [18], proliferation [19], hypoxia [20], and regulated cell death (RCD) [21] have been downloaded from previous studies. RCD included apoptosis, autophagy, anoikis, immunogenic cell death, pyroptosis, ferroptosis, and cuproptosis. The single sample gene set enrichment analysis (ssGSEA) methodology (“GSVA” R package) [22] was used to quantify the degree of enrichment of biological processes in a single sample based on its expression profiles. The relative invasion abundances of 28 immune cell types were also quantified using ssGSEA. Pearson’s correlation analysis was used to estimate the correlation between key genes and enrichment scores.

### 2.3. Tissue Samples and Immunohistochemistry (IHC)

A total of 97 tissue samples were collected from patients diagnosed with unresectable stage IIb–IIIc NSCLC who received radiotherapy, either alone or in combination with chemotherapy at the Affiliated Tumor Hospital of Harbin Medical University between June 2009 and January 2020. The study was sanctioned by the Medical Ethics Committee (KY2022‐57). Chest computed tomography scans were conducted on the patients 1 month after the completion of radiotherapy. According to the solid tumor efficacy evaluation criteria (RECIST 1.1), 3 patients were classified as complete response (CR) and 55 as partial response (PR), indicating radiosensitive, while 31 patients exhibited stable disease (SD), and 8 showed progressive disease (PD), were categorized as radioresistant. All the patients were recorded overall survival (OS), while 85 patients were recorded progression‐free survival (PFS). Here, OS was defined as the duration from diagnosis to death or last contact, whereas PFS was defined as the time from diagnosis to disease progression or death.

The tumor tissue sections from both human and murine specimens underwent deparaffinized in xylene followed by rehydration through a series of graded alcohol solutions, adhering to established procedures. Antigen retrieval was accomplished by immersing the slides in citrate buffer (0.01 M, pH 6.0), heating for 10 min, and allowed to cool naturally, thereby enhancing immunoreactivity. Subsequently, the slides were treated with 3% hydrogen peroxide for 15 min at ambient temperature. The specimens were then incubated overnight at 4°C with a primary antibody against SOD1 (1:100, rabbit polyclonal; Walleibio, China). Following this, the specimens were exposed to biotin‐labeled anti‐rabbit IgG goat secondary antibodies (1:200 Beyotime A0277, Haimen, China) for 30 min at room temperature, and then incubated with horseradish peroxidase‐labeled Avidin at 37°C for an additional 30 min. The slides were stained using DAB and subsequently counterstained with hematoxylin. Ultimately, each section underwent dehydrated via an alcohol gradient and covered with a coverslip.

The evaluation of staining outcomes was conducted by two independent pathologists with experienced in IHC assessment, who remained unaware of the associated clinical and pathological data. The scoring of staining results based to the cumulative intensity and proportion of positively stained cells. Staining intensity was classified into four categories: 0 indicating no staining, 1 representing light yellow or weak staining, 2 denoting yellow‐brown or moderate staining, and 3 signifying brown or strong staining. The proportion of positively stained tumor cells was scored as follows: 1 (1%–25% positive cells), 2 (26%–50% positive cells), 3 (51%–75% positive cells), or 4 (76%–100% positive cells). Final scores ranged from 0 to 7, with a median score of 2 utilized to differentiate between low and high SOD1 expression levels. Specimens scoring above 2 were categorized as high expression; while those scoring 2 or below were classified as low expression. Any discrepancies in scoring were resolved through discussion among two pathologists and a senior reviewer until a consensus was achieved among the senior pathologists.

### 2.4. Cell Transfection

Full‐length cDNA sequences of SOD1 were cloned in pcDNA3.1 (Youbio, Changsha, China, G105034), and Lipofectamine 3000 (Invitrogen, USA, L3000015) was used for transfection. A comprehensive description of the transfection procedure can be found in a prior publication [3]. In summary, cells were seeded in groups and cultured until reaching 80%–90% confluency. 7.5 μL Lipofectamine 3000:2.5 μg plasmid DNA were gently combined and incubated for 15 min at room temperature. The mixture was subsequently added dropwise to the wells containing the cell culture. The plate was then gently rocked to ensure uniform distribution and returned to the 37 °C, 5% CO_2_ incubator for subsequent experimental procedures. All steps were performed under sterile conditions. The pcDNA3.1 and primer were synthesized by the manufacturer’s instructions (GeneChem, Shanghai, China). The composition and detailed sequences are displayed in Table 1.

**Table 1:** PCR primer sequences and the sequences of SOD1 plasmids.

Name	Sequence (5′‐3′)
homo SOD1	F:GGTCCTCACTTTAATCCTCT
	R:CTTCATTTCCACCTTTGC
mus SOD1	F:GCAGGGAACCATCCACTT
	R:TGCCCAGGTCTCCAACAT
homo β‐actin	F:GGCACCCAGCACAATGAA
	R:TAGAAGCATTTGCGGTGG
mus β‐actin	F:CATCCGTAAAGACCTCTATGCC
	R:ATGGAGCCACCGATCCACA
pcDNA3.1‐Homo‐SOD1	ATGGCGACGAAGGCCGTGTGCGTGCTGAAGGGCGACGGCCCAGTGCAGGGCATCATCAATTTCGAGCAGAAGGAAAGTAATGGACCAGTGAAGGTGTGGGGAAGCATTAAAGGACTGACTGAAGGCCTGCATGGATTCCATGTTCATGAGTTTGGAGATAATACAGCAGGCTGTACCAGTGCAGGTCCTCACTTTAATCCTCTATCCAGAAAACACGGTGGGCCAAAGGATGAAGAGAGGCATGTTGGAGACTTGGGCAATGTGACTGCTGACAAAGATGGTGTGGCCGATGTGTCTATTGAAGATTCTGTGATCTCACTCTCAGGAGACCATTGCATCATTGGCCGCACACTGGTGGTCCATGAAAAAGCAGATGACTTGGGCAAAGGTGGAAATGAAGAAAGTACAAAGACAGGAAACGCTGGAAGTCGTTTGGCTTGTGGTGTAATTGGGATCGCCCAATAA

### 2.5. RNA Extraction and Real‐Time Fluorescence‐Based Quantitative PCR (RT‐qPCR)

The PCR methodology was performed in accordance with established techniques. Total RNA was extracted utilizing TRIpure (BioTeke, China, RP1001), followed by cDNA synthesis through the BeyoRT II M‐MLV reverse transcriptase (Beyotime, Jiangsu, China, D7160L). RT‐qPCR was executed using a BIOER TC‐96GH(b) PCR instrument (BIOER, Hangzhou, China), utilizing SYBR Green Master (ROX) (Solarbio, Beijing, China, SY1020). The optical density (OD) measurements of the target bands were analyzed using a gel‐pro analyzer. Detailed primer information can be found in Table 1. The fold change (FC) in gene expression levels was determined employing the 2^−ΔΔCt^ method [4].

### 2.6. Western Blotting

We adopted the methods of our previous study to perform western blotting analysis of SOD1 protein [4]. We used RIPA lysis buffer which including 1% phenylmethanesulfonyl fluoride (PMSF) to extract protein samples. The protein concentration in the lysates was determined by a protein BCA assay kit (Wanleibio WAL004, Shenyang, China). A total of 40 μg of protein per sample was subjected to separation via 10% sodium dodecyl sulfate (SDS) polyacrylamide gel electrophoresis, followed by polyvinylidene fluoride (PVDF) membranes. The membranes were incubated overnight 4°C with SOD1 antibody (1:500), and subsequently incubated with horseradish peroxidase‐labeled goat anti‐rabbit IgG antibody (1:1000). Detection of the bound antibodies was accomplished using an ECL western blot detection system. The quantification of SOD1 levels was performed via densitometric examination of the protein bands in relation to the total protein load, utilizing the Gel‐Pro Analyzer software. β‐actin utilized as a loading control, with all antibodies sourced from Wanlei bio. The visualization of protein bands was visualized using enhanced chemiluminescence (Wanleibio, China, WLA003), followed by quantification using a Gel image processing system (Gel‐Pro‐Analyzer software).

### 2.7. Cell Proliferation Assay

Cell viability was determined utilizing the cell counting kit‐8 (CCK‐8; Wanleibio, WLA074), following the protocol outlined by the manufacturer. In brief, cells were seeded in 96‐well plates at a concentration of 3 × 10^3^ cells per well and incubated for periods of 24, 48, 72, or 96 h. At the designated time intervals, each well received 10 μL CCK‐8 solution followed by a 2‐h incubation. The absorbance was subsequently measured at 450 nm using a microplate reader (BIOTEK, Vermont, USA, 800TS). Cell proliferation curves were constructed by analyzing the absorbance of the OD readings at 450 nm across various time intervals. The assay was performed with five replicates for each sample, and three independent experiments were conducted to ensure reliability.

### 2.8. Flow Cytometry Assay

The assessment of apoptosis was conducted utilizing an Annexin V‐FITC/PI Kit (Wanleibio, Shenyang, China, WLA001). The methodology has been comprehensively outlined in a preceding publication. In summary, cells of each group were thoroughly mixed with 5 μL AnnexinV‐FITC, and then with 10 μL propidium iodide. Subsequently, the mixture was incubated at ambient temperature, protected from light for a duration of 15 min. Finally, the flow‐through was detected using a NovoCyte cytometer (Agilent, Santa Clara, CA, USA). The result was processed with FlowJo software.

### 2.9. Cell Invasion and Migration Assay

Cells were seeded in a serum‐free medium within the upper compartment of a transwell insert featuring an 8‐μm pore size (LABSELECT, Anhui, China, 14341). For the invasion assays, the membrane was pre‐treated with 40 μL Matrigel (Corning, USA) to establish a matrix barrier. While for migration assays, Matrigel was omitted. The lower compartment was supplied with a standard medium that contained 10% FBS. Following incubation at 37°C for 24 h, the cells that had traversed to the lower surface of the membrane were fixed with 4% paraformaldehyde for a duration of 30 min, subsequently stained with 0.5% crystal violet, and photographed under a light microscope. Cell counts were performed at 200× magnification across five random fields per well using a light microscope. Each experiment was conducted in triplicate.

### 2.10. Irradiation

Cells were treated with RBMD for 18 h before receiving 8 Gy of radiation. The radiation parameters were 6 MV X‐rays with a source‐tumor distance of 100 cm. The cell culture dish was thoroughly cleaned with film before radiation treatment to prevent cell contamination. Following the radiotherapy, cells were cultured for an additional 24 h under standard conditions.

### 2.11. Single‐Cell Gel Electrophoresis

Cells were collected and subsequently combined with low melting point agarose at a concentration of 2%, maintaining a 1:1 volume ratio. The mixture was then spread onto a Petri dish previously coated with a layer of 0.5% standard melting point agarose, and refrigerated at 4°C until solidification. Once solidified, the cell‐containing agarose layer was immersed in lysis solution (pH 8) and left overnight at 4°C. The cells were undergoing three washing cycles with precooled double‐distilled water (20 min each) and incubated in precooled electrophoresis buffer for 45 min. Electrophoresis was then performed at 35 V for 35 min. Cells were then stained with a fluorescent dye solution for 15 min, rinsed three times with water (each rinse lasting 5 min), and photographed under a fluorescence microscope.

### 2.12. In Vivo Experiments

C57BL/6 male mice, aged 4–6 weeks, with 200 μL of lung cancer cell (LLC) suspension (~2 × 10^6^ cells). Upon reaching a tumor volume of 200 mm^3^, the mice were treated with local radiotherapy (4 Gy/dose) every 3 days for five courses, resulting in a cumulative dose of 20 Gy. According to equivalent measurements of the body surface of humans and animals, mice were categorized into distinct groups (*n* = 3 mice per group): high‐dose (treated with two times the equivalent dose of RBMD, 32.5 g/kg), medium‐dose (treated with an equivalent dose of RBMD, 16.25 g/kg), low‐dose (treated with 0.5 times the equivalent dose of RBMD, 8.125 g/kg), irradiation (IR) (0 g/kg RBMD), and control (nonirradiated (non‐IR), untreated mice) groups. The changes in tumor size and volume among the individuals of the five groups were observed for 42 days. All mice were sacrificed 24 h after the last dose and tumor tissues were collected for weight measurement and subsequent experiments.

β‐Thy was administrated to mice through intraperitoneal injection at a dose of 500 mg/kg/12 h [23], at the same time as radiotherapy was started, and was given continuously for 18 days, and the mice in this group were sacrificed 24 h after the last.

### 2.13. Liquid Chromatography‐Tandem Mass Spectrometry

The quantification of β‐thymidine (β‐Thy) in RBMD samples was carried out utilizing through the application of liquid chromatography coupled with tandem mass spectrometry (LC‐MS/MS). RBMD samples were ultrasonicated for 1 h, combined at a 1:1 (v/v) proportion with 80% methanol (500 μL). This mixture was thoroughly homogenized through vertexing and subsequently subjected to centrifugation at 13,000 rpm for 10 min at a temperature of 4°C. Each supernatant was then injected into the LC‐MS/MS system for analysis. The analytical process employed a TSQ Quantum triple quadrupole mass spectrometer (Thermo Fisher Scientific, USA) in conjunction with an UltiMate 3000 RS chromatograph (Thermo Fisher Scientific, USA). The acquisition of chromatograms and the integration of the analytes were executed using the Xcalibur software (Thermo Fisher Scientific, USA), while linear regression analysis was conducted with a weighting factor of 1/*x* ^2^.

### 2.14. Statistical Analysis

The experimental data was undergoing analysis through specialized statistical software such as SPSS 25, GraphPad Prism version 9.0 or R version 4.2.1. The mean ± standard difference of the results obtained in three independent experiments was calculated for all data obtained in the experiment. A Student’s *t*‐test was employed to compare the means of the two samples. For the different expression and functional enrichment analyses, the *p*‐value has been modified using the Benjamin–Hochberg procedure for several tests to control the false discovery rate (FDR). The 3‐year survival rate of the patients served as the primary endpoint of interest, with patients exceeding a 3‐year follow‐up being censored at the 3‐year mark. Survival curves were generated using the Kaplan–Meier method and statistically compared using the log‐rank test. A univariate Cox regression model was used to analyze the relationships between the gene and the 3‐year survival rate of the patients. Differences with two‐sided *p*  < 0.05 or FDR < 0.05 for multiple tests were considered statistically significant.

## 3. Results

### 3.1. RBMD Inhibits the Growth of Tumors Transplanted in Mice

**Figure 1:**
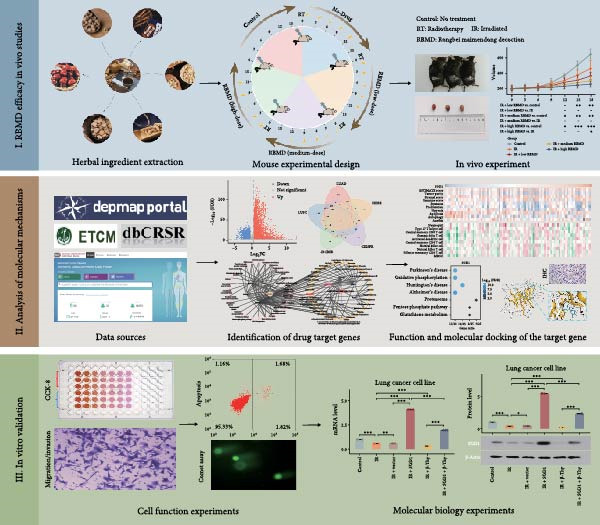
Flowchart of this study.

The flowchart of this study is displayed in Figure 1.

The in vivo experimental results (Figure 2) indicated that, in comparison with the control group, the tumors in treated mice exhibited a significant reduction in size and weight following both irradiation and RBMD treatment, with the high‐dose group showing the most pronounced reduction in tumor size and weight compared to other groups (Student’s *t*‐test, *p*  < 0.05, Figure 2b). Statistically significant differences in tumor size were recorded among the groups (Student’s *t*‐test, *p*  < 0.05, Figure 2c).

Figure 2RBMD inhibits the growth of tumors from transplanted cancer cells in mice. (a) Schematic representation of treatment regimes. (b) The tumor size and weight of mice subjected to different treatment. (c) Tumor growth kinetics (*n* = 3 mice per group). Tumor volume data are presented as mean ± SEM. Statistical significance was determined using a two‐tailed *t*‐test.  ^∗^ *p*  < 0.05;  ^∗∗^ *p*  < 0.01;  ^∗∗∗^ *p*  < 0.001.

**Figure:**
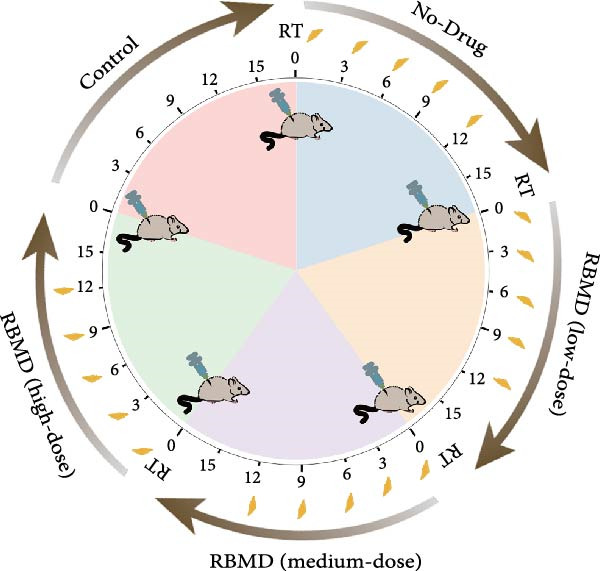
(a)

**Figure:**
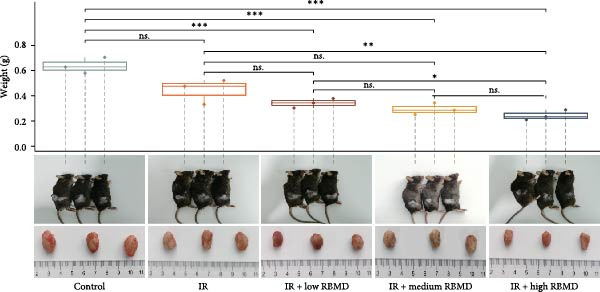
(b)

**Figure:**
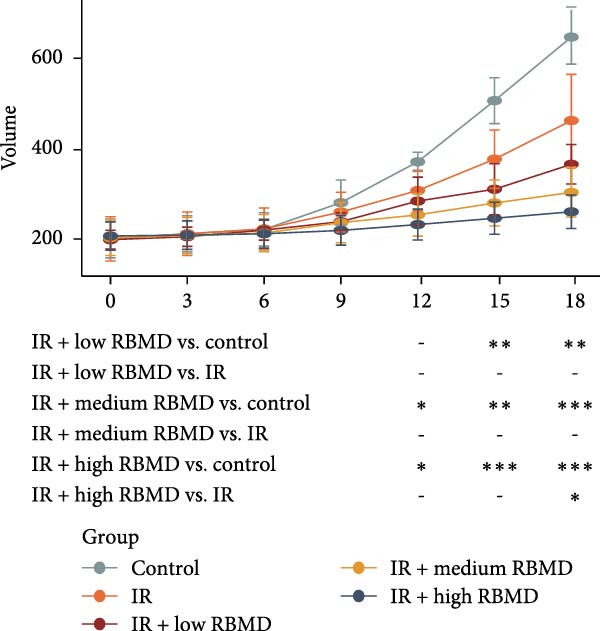
(c)

### 3.2. Identification of RBMD Key Target Genes Involved in NSCLC Radiosensitivity

Our initial analyses of the data retrieved from TCGA led to the identification of 10,760 and 11,966 DEGs in LUAD and LUSC cells, respectively, compared to normal tissue (Student’s *t*‐test, FDR < 0.05, Figure 3a, b). We then screened 693 essential genes related to NSCLC cell survival whose CERES scores were less than −1 in more than 75% of the NSCLC cell lines in the CRISPR‐Cas9 screening dataset (DepMap portal, https://depmap.org/portal/download/) [24]. Finally, we identified 19 essential DEGs related to radiosensitivity, annotated in dbCRSR (http://www.xialab.info:8080/dbCRSR/index.jsp) [25], five of which were targets of compounds in RBMD, including *AURKB*, *BIRC5*, *PLK1*, *RAD51*, and *SOD1* (Figure 3c).

Figure 3Identification of the key target genes of RBMD related to NSCLC radiosensitivity. (a) Volcano plot representing differentially expressed genes (DEGs) between LUAD and normal samples from a TCGA dataset. (b) Volcano plot depicting DEGs in LUSC and normal samples from a TCGA dataset. (c) Venn diagram illustrating the intersections among DEGs in LUAD and LUSC samples, essential genes related to NSCLC cell survival (CRISPR), radiotherapy‐related genes (dbCRSR), and RBMD‐related genes (HERB). (d) Interaction network linking RBMD and its components with functional genes. Dark pink: RBMD formula; purple: herbs in RBMD; yellow: isolated RBMD compounds, green: RBMD target genes; light pink: gene functions.

**Figure:**
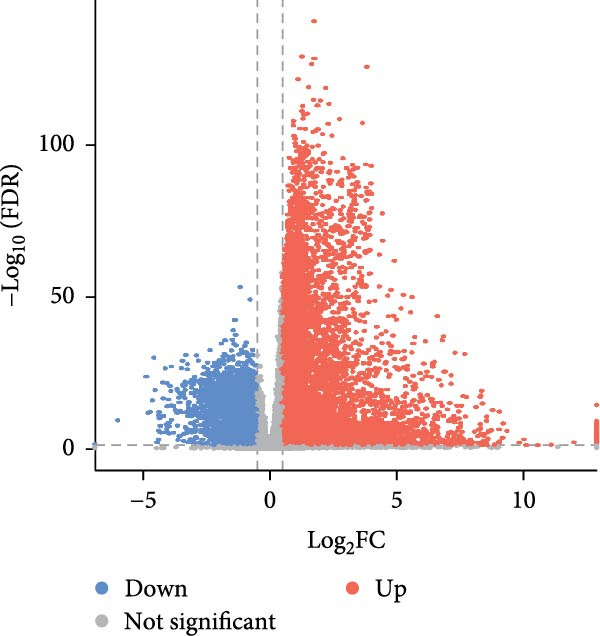
(a)

**Figure:**
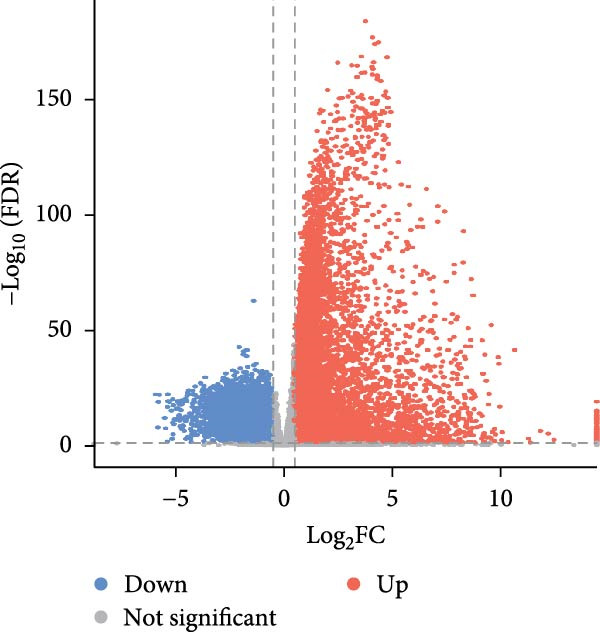
(b)

**Figure:**
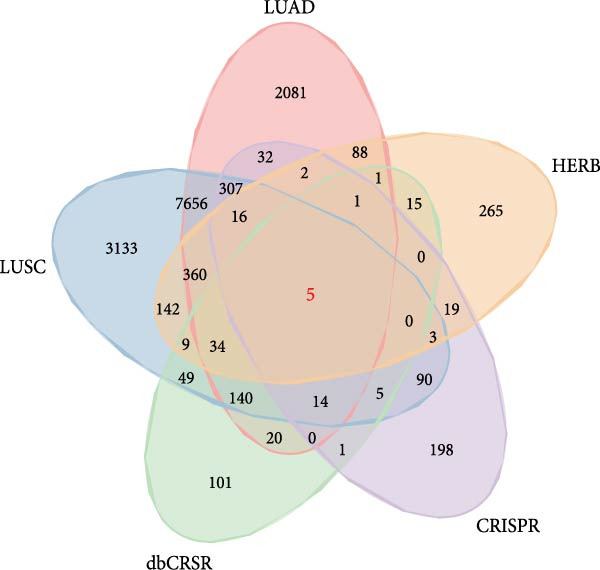
(c)

**Figure:**
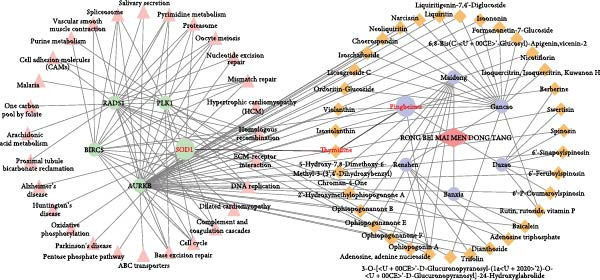
(d)

We further identified a set of genes that significantly correlated with *AURKB*, *BIRC5*, *PLK1*, *RAD51*, and *SOD1* (Pearson correlation, |*r*| > 0.5, FDR < 0.05), and found that these genes were significantly enriched in pathways related to radiosensitivity, including DNA repair pathways (such as mismatch repair and base excision repair) and oxidative phosphorylation (FDR < 0.05, Supporting Information 1: Figure S1). Based on these results, we constructed a network linking the six herbs present in RBMD with the five target genes of RBMD (Figure 3d). Notably, in the network, β‐Thy, a component of RBMD, could target *SOD1*. The mRNA expression differences of *SOD1* between cancer samples and normal controls in the TCGA‐LUAD and TCGA‐LUSC cohorts are shown in Supporting Information 2: Figure S2. Our previous research demonstrated that high expression of SOD1 could induce radiotherapy resistance by reducing ROS accumulation after radiotherapy [3]. Therefore, we infer that one mechanism through which RBMD enhances radiosensitivity is the inhibition of SOD1 by the β‐Thy component.

### 3.3. RBMD Contains β‐Thy in Regulating *SOD1* Expression

Bioinformatics analyses indicated a significant positive correlation between *SOD1* expression and stemness and proliferation scores, while it exhibited a negatively association with the infiltration of multiple immune cells, including natural killer T cells (Pearson correlation, FDR < 0.05, Figure 4a). These finding underscores demonstrating the molecular and immunological roles of *SOD1* in promoting cancer progression. Furthermore, our analysis indicated that elevated expression of *SOD1* had an inhibitory effect on multiple regulatory cell death pathways, such as apoptosis and immunogenic cell death (Pearson correlation, FDR < 0.05, Figure 4a). The functional enrichment results for *SOD1* showed that its correlated genes were significantly enriched in pathways pertinent to radiosensitivity (FDR < 0.05, Figure 4b), such as oxidative phosphorylation, the proteasome, the pentose phosphate pathway and glutathione metabolism. Moreover, the differential analysis of two independent datasets confirmed that *SOD1* was significantly high expression in NSCLC samples when compared with adjacent normal samples in GSE19804 (NSCLC vs. normal, 60 vs. 60, *t* = 5.94, *p*  < 0.0001, Figure 4c) and in GSE32863 (NSCLC vs. normal, 58 vs. 58, *t* = 2.86, *p*  < 0.0001, Figure 4c). To determine the correlation between radiation resistance and *SOD1*, we collected 97 NSCLC patients undergoing radiation therapy. Representative immunohistochemical staining images depicting SOD1 protein expression in radioresistant and radiosensitive samples are illustrated in Figure 4d. The staining scores of SDO1 were significantly greater in the 39 radioresistant samples than in the 58 radiosensitive samples (Student’s *t*‐test, *p*  < 0.0001; Figure 4e). Survival analysis demonstrated that patients classified in the high‐SOD1 group had significantly shorter 3‐year OS and 3‐year PFS than that of patients in the low‐SOD1 group (3‐year OS: log‐rank *p*  < 0.0001, HR = 2.29, 95% CI = 1.54–3.42, Figure 4f; 3‐year PFS: log‐rank *p* = 0.0041, HR = 1.56, 95% CI = 1.02–2.38, Figure 4g).

Figure 4Functional analyses of SOD1 and its independent validation. (a) Participation of SOD1 in a variety of metabolic and immune pathways, including stemness, proliferation, hypoxia, regulatory cell death, immune scores, and the infiltration proportions of immune cells. (b) KEGG enriched pathways of the key genes correlated with SOD1 expression. (c) The boxplot of SOD1 mRNA expression between NSCLC and pair normal controls in two public independent datasets (GSE19804 and GSE18842). (d) Immunohistochemistry images showcasing the SOD1 protein in radioresistant versus radiosensitive NSCLC samples. (e) Notable differences in SOD1 protein expression level between radioresistant and radiosensitive NSCLC samples. (f–g) Kaplan–Meier survival curves of NSCLC tumor specimens stratified based on SOD1 protein levels as detected through immunohistochemistry.

**Figure:**
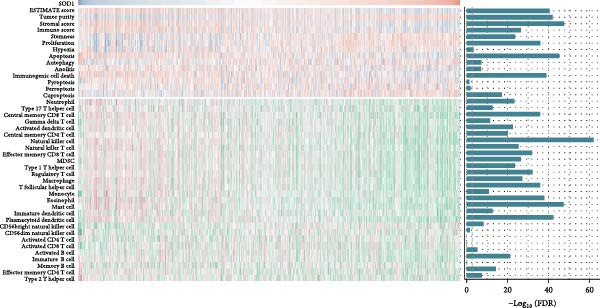
(a)

**Figure:**
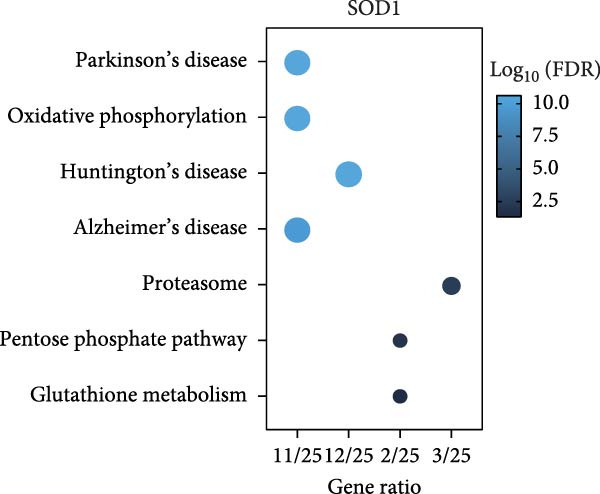
(b)

**Figure:**
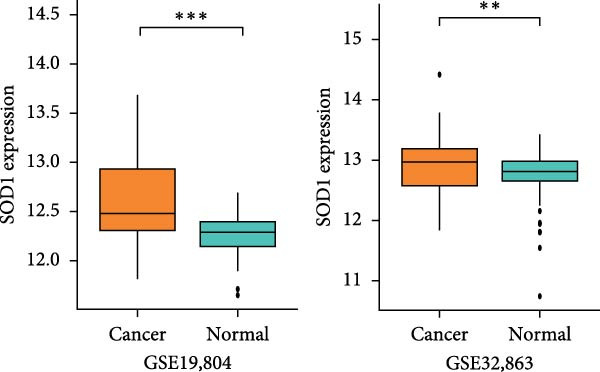
(c)

**Figure:**
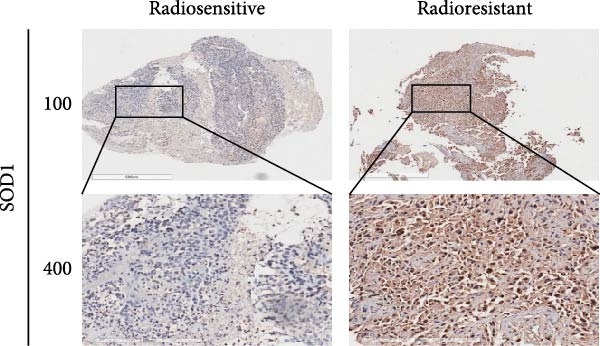
(d)

**Figure:**
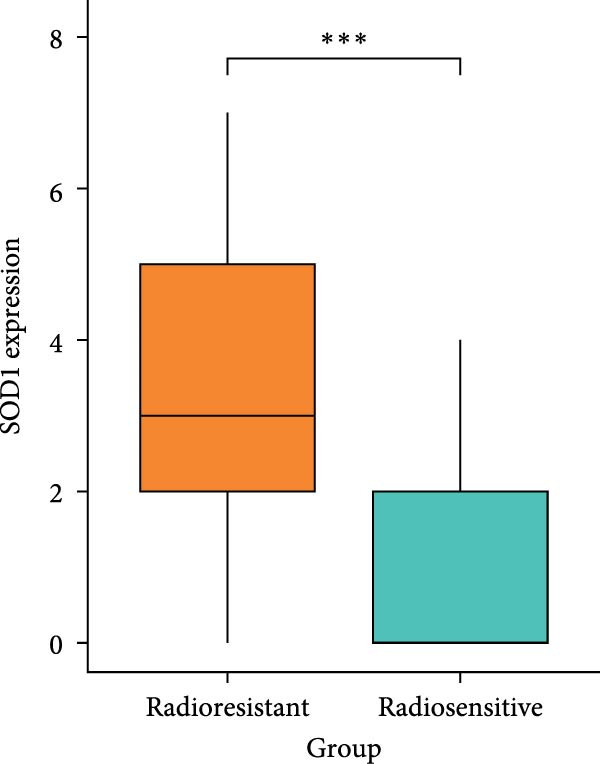
(e)

**Figure:**
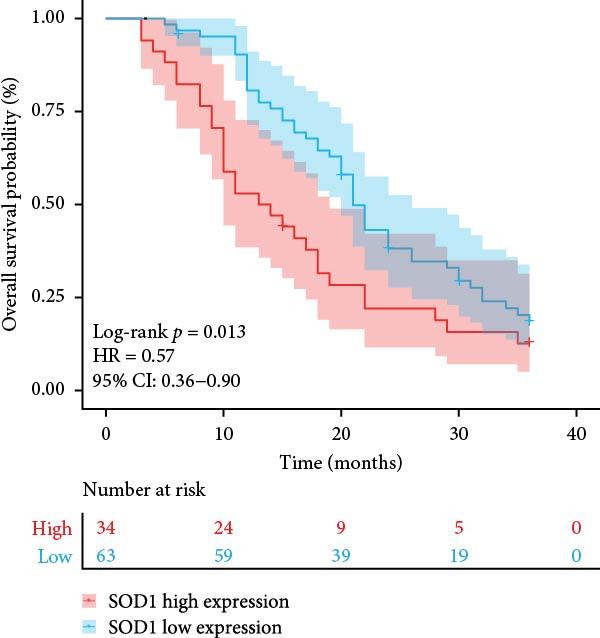
(f)

**Figure:**
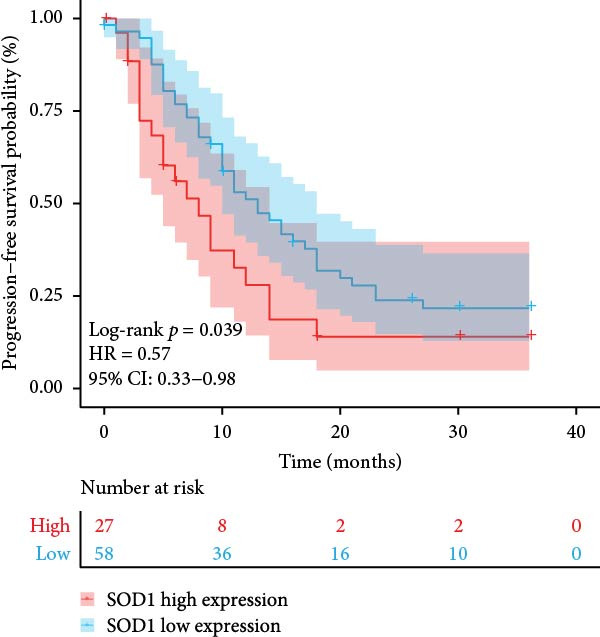
(g)

Subsequently, molecular docking simulations between thymidine and SOD1, using the CB‐Dock2 server (https://cadd.labshare.cn/cb-dock2/php/index.php) [26], showed that they had high docking scores (Vina score < −7 kcal/mol) (Figure 5a). These results indicate that thymidine in RBMD may achieve radiotherapy sensitization by inhibiting SOD1 expression. For determining the concentration of β‐Thy in RBDM, a standard curve was obtained with a good linearity within a 20–5000 ng/mL range by LC‐MS/MS (Figure 5b). According to our data, RBMD can inhibit tumor growth (due to cancer cell transplantation) in mice exposed to radiation in a dose‐dependent manner.

Figure 5The correlation of SOD1 with β‐Thy. (a) Molecular docking simulation between SOD1 and thymidine. The Vina score for this molecular docking was 7.5, showing a strong docking activity. (b) Standard curve constructed for determining RBMD concentrations in tumor sample tissues from mice. (c) IHC images and (d) concentration of SOD1 in tumor tissues from mice (after cancer cell transplantation, irradiation, and RBMD treatment). Data represent the means ± SEM. Statistical significance was determined via two‐tailed Student’s *t*‐tests.  ^∗^ *p*  < 0.05;  ^∗∗^ *p*  < 0.01;  ^∗∗∗^ *p*  < 0.001.

**Figure:**
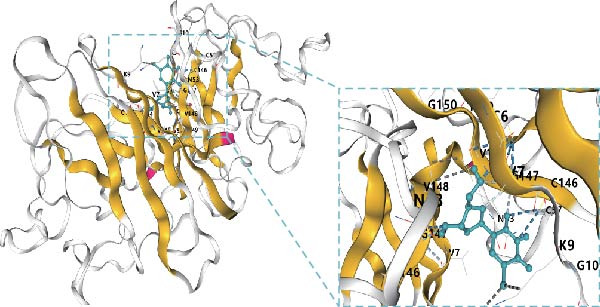
(a)

**Figure:**
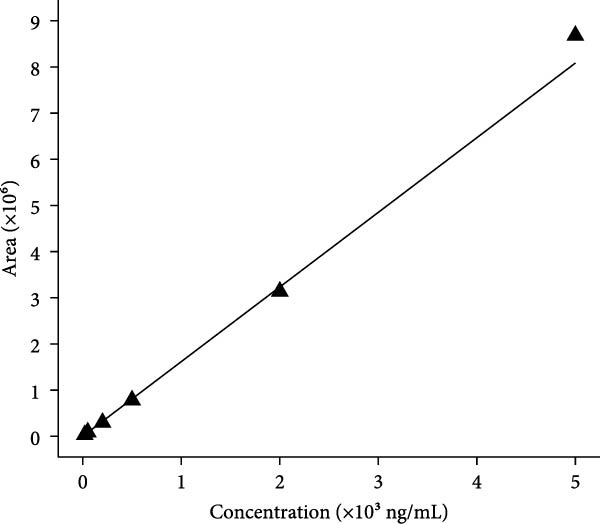
(b)

**Figure:**
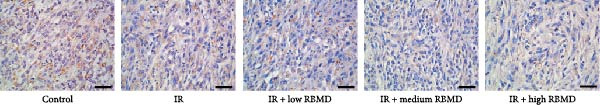
(c)

**Figure:**
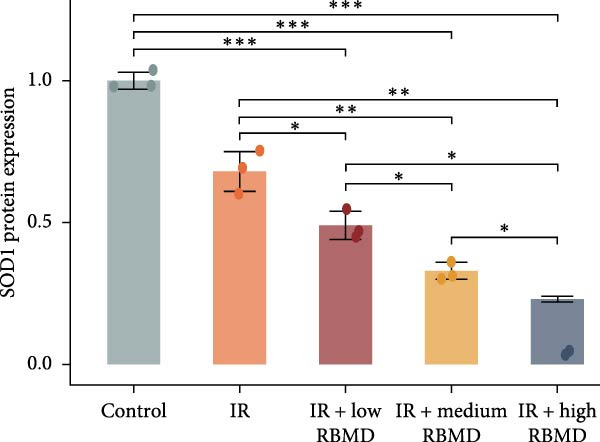
(d)

The RT‐qPCR analysis of mouse tumor tissues revealed a concentration‐dependent decrease in *SOD1* mRNA expression with increasing RBMD (Student’s *t*‐test, *p*  < 0.05; Figure 5c). Consistently, the IHC results confirmed a significant reduction in SOD1 protein expression (Student’s *t*‐test, *p*  < 0.05; Figure 5d). Therefore, RBMD enhanced the radiosensitivity of NSCLC cells by inhibiting the expression of SOD1 after radiotherapy.

### 3.4. β‐Thy Inhibits the High Expression of SOD1, at Both mRNA and Protein Levels, in Lung Cancer Cells

Compared with those in normal bronchial epithelial cell lines, SOD1 mRNA and protein levels were found to be significantly elevated in lung cancer cell lines (Figure 6a, b). Given that A549 and H1650 showed moderate SOD1 expression levels, these cell lines were selected for subsequent experiments. After treatment with β‐Thy, SOD1 mRNA and protein expression were suppressed in both A549 and H1650 cells, and the difference with that of normal cell lines was statistically significant (A549: Student’s *t*‐test, *p*  < 0.0001, Figure 6c; H1650: Student’s *t*‐test, *p*  < 0.0001, Figure 6d).

Figure 6β‐Thymidine inhibits high expression of SOD1 at both mRNA and protein levels in lung cancer cells. SOD1 (a) mRNA and (b) protein levels in different human cell lines. The above protein panel shows the protein expression of SOD1 in different human cell lines, and the below panel shows the protein expression of β‐actin in the same cell lines. SOD1 (c) mRNA and (d) protein expression in A549 and H1650 cells treated with β‐thymidine. The above protein panel shows the protein expression of SOD1 in A549 and H1650 cells treated with β‐thymidine, and the below panel shows the protein expression of β‐actin in the same cell lines. (e–h) mRNA and protein expression of SOD1 in controls, cells overexpressing SOD1 and cells treated with β‐thymidine in A549 and H1650 cell, respectively. The above protein panels show the protein expression of SOD1 in A549 (f) and H1650 (h) cells treated with β‐thymidine and overexpressing SOD1, respectively, and the below panel shows the protein expression of β‐actin in the same cell lines. Data represent the means ± SEM. Statistical significance was analyzed through a two‐tailed Student’s *t*‐test, with significance levels indicated as  ^∗^ *p* < 0.05;  ^∗∗^ *p* < 0.01;  ^∗∗∗^ *p* < 0.001.

**Figure:**
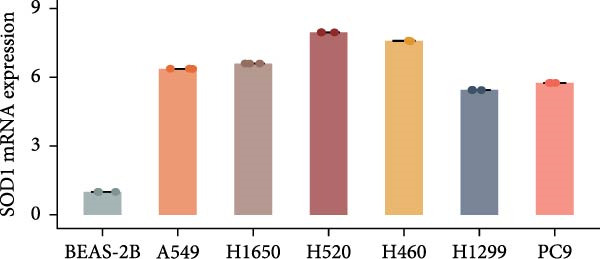
(a)

**Figure:**
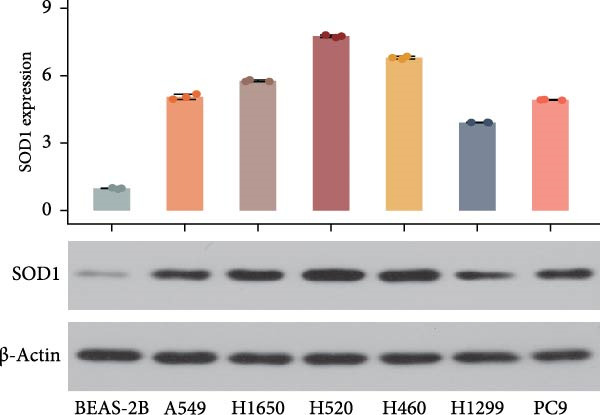
(b)

**Figure:**
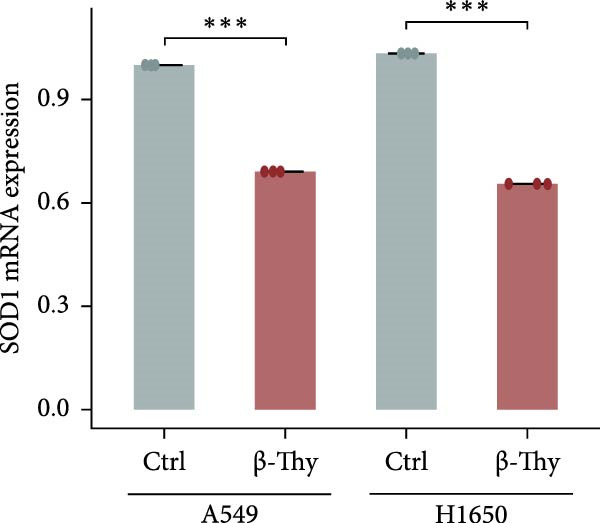
(c)

**Figure:**
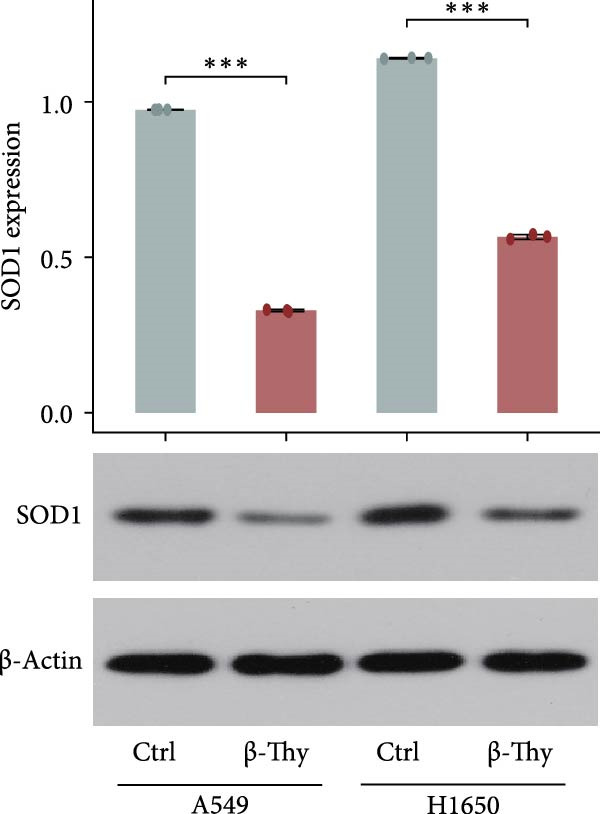
(d)

**Figure:**
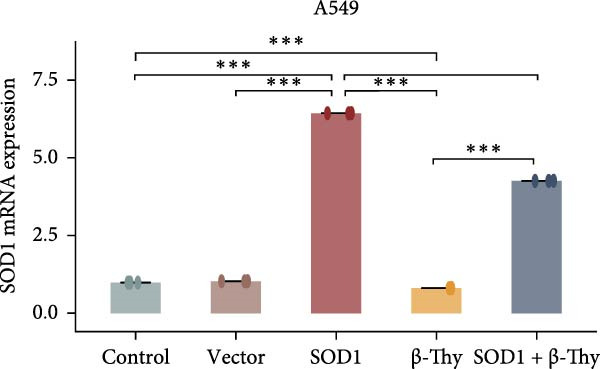
(e)

**Figure:**
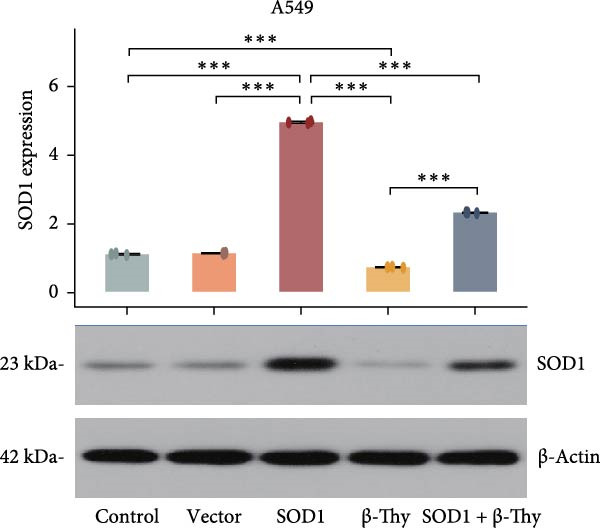
(f)

**Figure:**
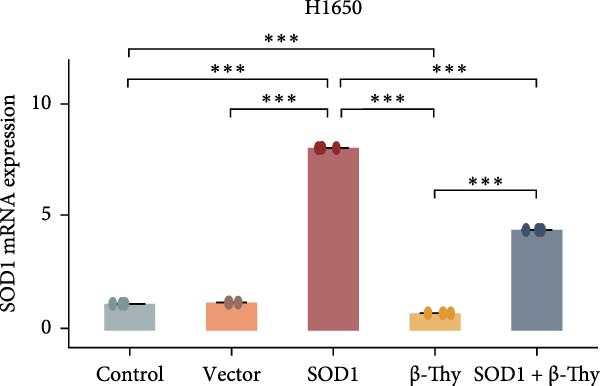
(g)

**Figure:**
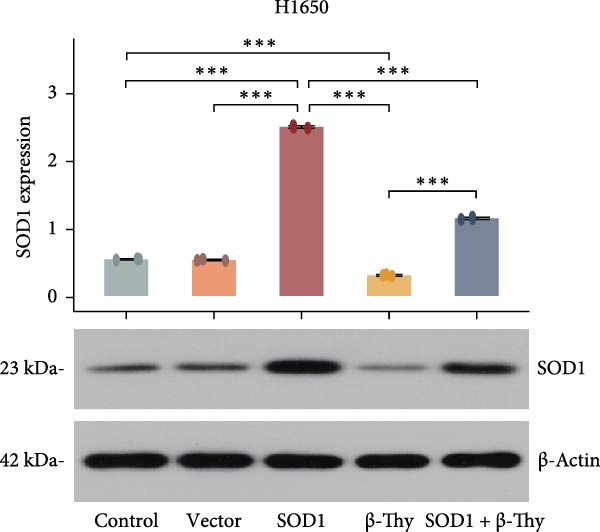
(h)

We constructed a human SOD1 (NM_000454.5, CDS = 465 bp) overexpression vector and used it to transfect A549 and H1650 cells in logarithmic phase. Cells transfected using empty vectors serving as controls. Notably, the mRNA and protein expression levels of SOD1 exhibited substantial elevation in A549 and H1650 cells transfected with the SOD1 overexpression vector (Student’s *t*‐test, *p*  < 0.05, Figure 6e–h). Interestingly, β‐Thy addition to A549‐ and H1650‐SOD1 overexpressing cell lines led to a significant reduction in SOD1 expression levels (Student’s *t*‐test, *p*  < 0.05, Figure 6e–h).

### 3.5. β‐Thy Suppressed SOD1 Expression and Enhanced Radiosensitivity of NSCLC Cells

CCK‐8 assay results revealed that SOD1 overexpression significantly augmented the proliferative capacity of A549 and H1650 cells compared to control cell lines, and the ability was significantly weakened by adding β‐Thy to the culture medium (Student’s *t*‐test, *p*  < 0.05, Figure 7a, b). This suggests that β‐Thy effectively counteracts the proliferative advantage conferred by SOD1 in lung cancer cells.

Figure 7In vitro experiments, β‐thymidine affected the malignant phenotype of NSCLC cells by inhibiting the expression of SOD1. Cell viability (a,b), invasion (c,e), migration (d,f), and apoptosis (g,h) of A549 and H1650 cells, respectively, treated with β‐thymidine and overexpressing SOD1. Data represent the means ± SEM. Statistical significance was determined using a two‐tailed Student’s *t*‐test.  ^∗^ *p*  < 0.05;  ^∗∗^ *p*  < 0.01;  ^∗∗∗^ *p*  < 0.001.

**Figure:**
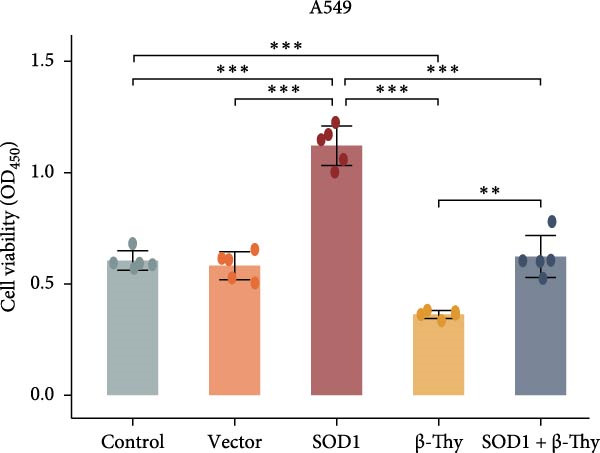
(a)

**Figure:**
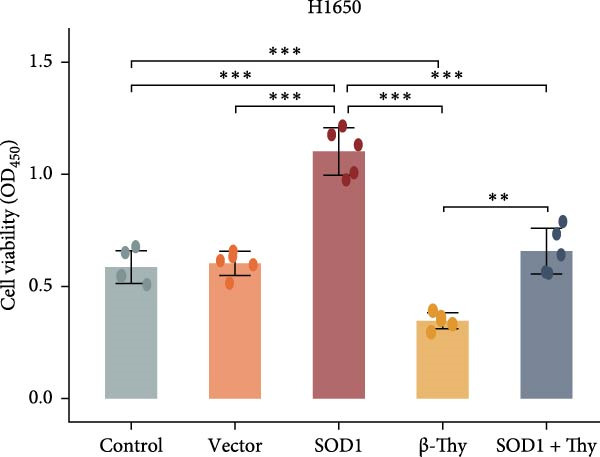
(b)

**Figure:**
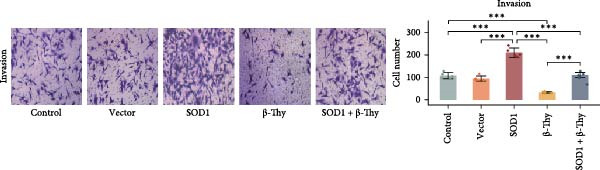
(c)

**Figure:**
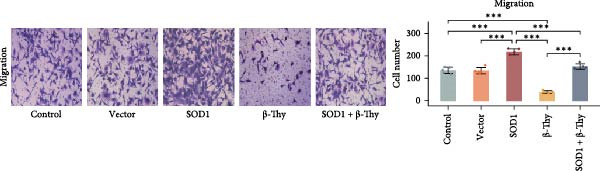
(d)

**Figure:**
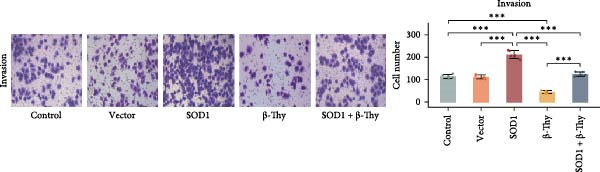
(e)

**Figure:**
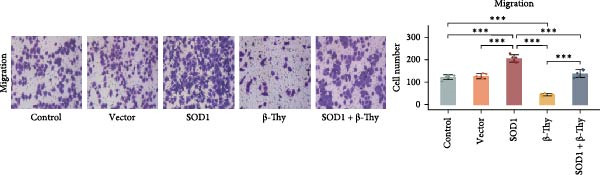
(f)

**Figure:**
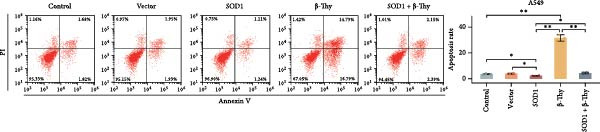
(g)

**Figure:**
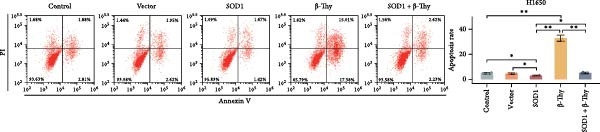
(h)

Additionally, transwell assay results indicated that SOD1 overexpression enhanced both the migratory and invasive capabilities of A549 and H1650 cells. However, these capacities were significantly reduced following the addition of β‐Thy to the medium (Student’s *t*‐test, *p*  < 0.05, Figure 7c–f), further demonstrating that β‐Thy was able to reverse the effect of SOD1 on cell migration and invasion. Additionally, apoptosis level was significantly reduced in A549 and H1650 cells overexpressing SOD1, but significantly enhanced after adding β‐Thy to the medium (Student’s *t*‐test, *p*  < 0.05, Figure 7g, h), indicating that β‐Thy was able to reverse the inhibitory effect of SOD1 on apoptosis.

### 3.6. β‐Thy Reduces SOD1 Expression and Promotes DNA Damage in NSCLC Cells After Radiotherapy

To further investigate the mechanism by which β‐Thy affects NSCLC radiosensitivity, we first subjected A549 and H1650 cells transfected with the SOD1 overexpression vector (IR + SOD1 group) or treated with β‐Thy (IR + β‐Thy group) to X‐ray irradiation. In comparison to the control (non‐IR) group, SOD1 expression was elevated in the IR group, but reached its highest levels in the IR + SOD1 group and its lowest levels in the IR + β‐Thy group. Notably, SOD1 expression levels in the IR + SOD1 + β‐Thy group were in between those of the last two groups, indicating that the high SOD1 expression levels induced by irradiation in NSCLC cells could be significantly reduced by adding β‐Thy to the medium (Student’s *t*‐test, *P*  < 0.05, Figure 8a–d).

Figure 8β‐Thymidine reduces SOD1 expression, promoting DNA damage, in NSCLC cells after radiotherapy. SOD1 mRNA (a,c) and protein (b,d) expression in A549 and H1650 cells treated with β‐thymidine and carrying a SOD1 overexpression vector after exposure to ionizing radiation (IR). The above protein panel shows the protein expression of SOD1 in A549 cells (b) and H165 cells (d) treated with β‐thymidine and carrying a SOD1 overexpression vector after exposure to ionizing radiation (IR), and the below panel shows the protein expression of β‐actin in the same cell lines. DNA damage was quantified via single‐cell gel electrophoresis by measuring tail lengths (e,f, left) and tail DNA damage (right). (g) The tumors size and weight of mice subjected to different treatments. (h) Tumor growth kinetics (*n* = 3 mice per group). Data represent the means ± SEM. Statistical significance was determined using a two‐tailed Student’s *t*‐test.  ^∗^ *p* < 0.05;  ^∗∗^ *p* < 0.01;  ^∗∗∗^ *p* < 0.001.

**Figure:**
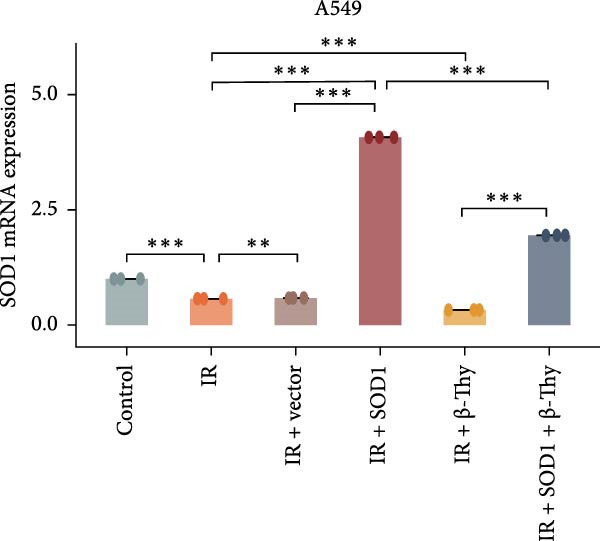
(a)

**Figure:**
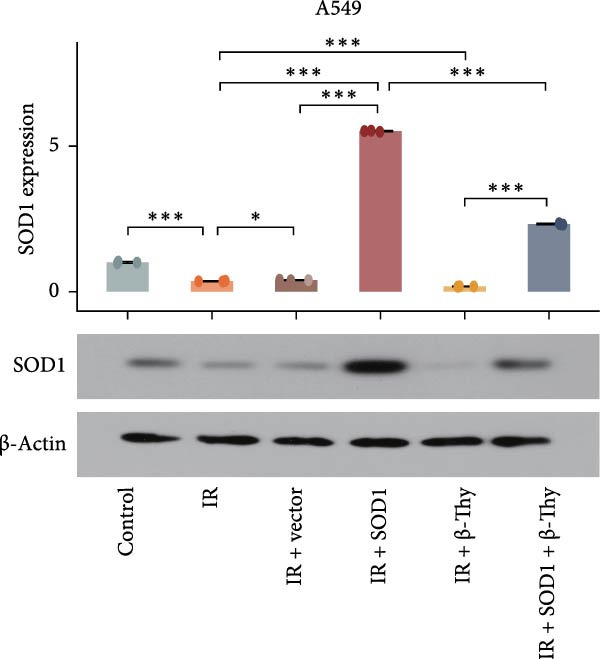
(b)

**Figure:**
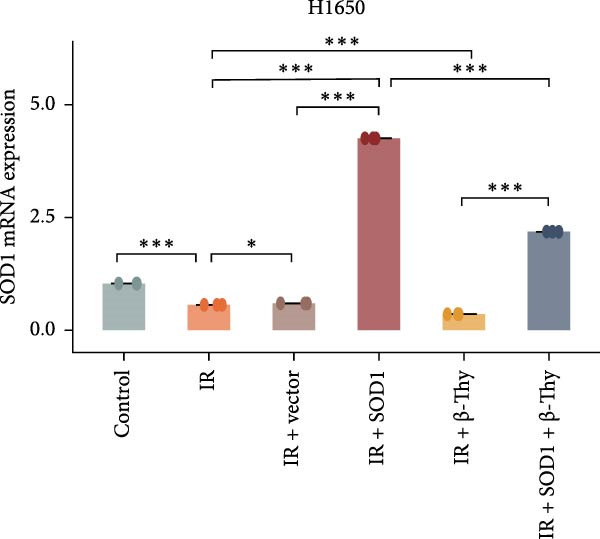
(c)

**Figure:**
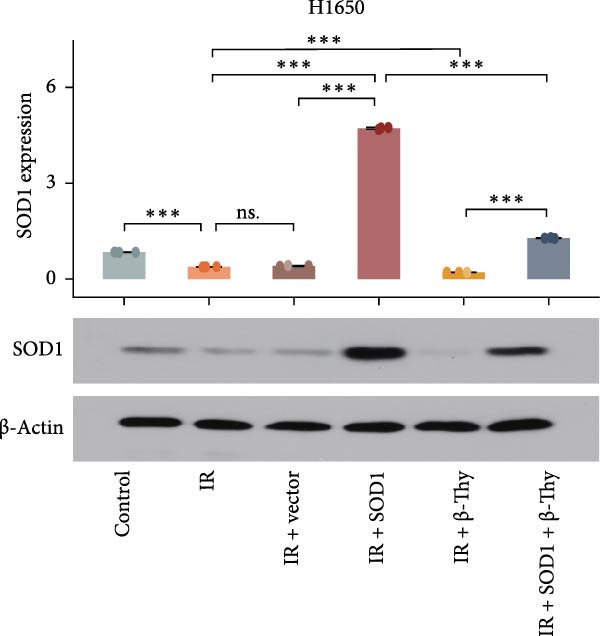
(d)

**Figure:**
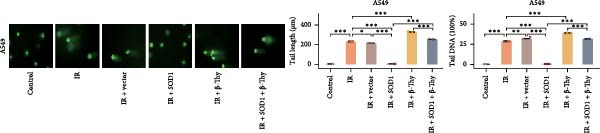
(e)

**Figure:**
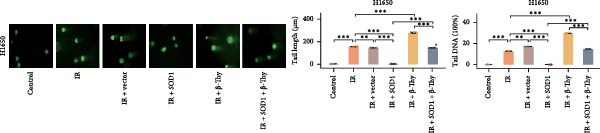
(f)

**Figure:**
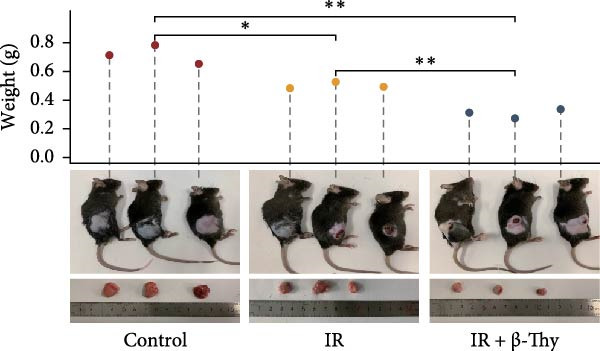
(g)

**Figure:**
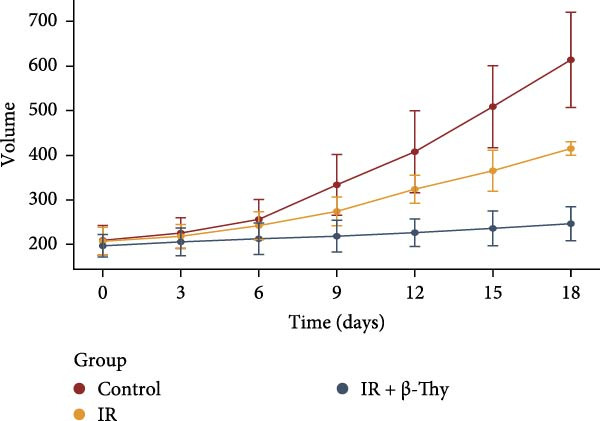
(h)

DNA damage can be detected by single‐cell gel electrophoresis when double‐strand breaks occur in intracellular DNA, with the severity assessed by the percentage of DNA content in the comet tail. The electrophoresis results indicated the percentage of DNA within the comet tail increased in all cell groups after irradiation, with the lowest percentage of DNA in the comet tails of cells in the IR + SOD1 group, the highest percentage of DNA in the comet tails of cells in the IR + β‐Thy group, and an in‐between DNA percentage in the comet tails of cells in the IR + SOD1 + β‐Thy group, indicating that the addition of SOD1 reduced DNA damage due to radiation, while the addition of β‐Thy increased such DNA damage. DNA damage caused by X‐rays in NSCLC cells overexpressing SOD1 and cultured in a β‐Thy‐containing medium was significantly greater than that observed in the IR + SOD1 group and significantly less than that in the β‐Thy group (Student’s *t*‐test, *p*  < 0.05, Figure 8e, f).

### 3.7. β‐Thy Inhibits the Growth of Transplanted Tumors in Mice

To validate the radiosensitizing effect of β‐Thy in vivo experiments, we established β‐Thy alone group and compared it with the IR alone group and the control group, respectively. The results revealed that in comparison to the control groups, the tumors size and weight of β‐Thy‐treated mice were obviously reduced, consistent with the effects of high‐dose RBMD treatment (Student’s *t*‐test, *p*  < 0.05, Figure 8g). The differences of tumor volumes among the groups were statistically significant (Student’s *t*‐test, *p*  < 0.05, Figure 8h).

## 4. Discussion

Previous investigations have indicated that a range of bioactive compounds in TCM remedies, such as artemisinin and ginseng saponins, may hold the potential to enhance patient outcomes, increase sensitivity to radiotherapy, and reduce radiotherapy‐induced toxicity [27–29]. Furthermore, studies have underscored the efficacy of Maimendong Tang in alleviating adverse reactions to radiotherapy in individuals with malignant tumors, demonstrating substantial therapeutic advantages [8]. However, these investigations have predominantly concentrated on the effects of radiotherapy and the therapeutic efficacy of TCM interventions, with relatively limited exploration into the mechanisms by which TCM enhances radiosensitivity in lung cancer cells. Our previous studies revealed that red bean cedar is the active ingredient in maitake Maimendong Tang, and that it can enhance chemotherapeutic efficacy by inhibiting proliferation, inducing apoptosis, and reversing drug resistance in NSCLC cells through the activation of multiple pathways. Additionally, our previous findings revealed that, following radiotherapy, mice transplanted with NSCLC cells and treated with varying concentrations of *Phellodendron* bark soup exhibited a significant radiosensitizing effect, suggesting the potential role of certain constituents in this *Phellodendron* bark soup in enhancing the radiosensitivity of NSCLC cells.

Investigations employing bioinformatics have unveiled that pingbeimu, an essential element of Fuzibei Maimendong Tang, encompasses a broad spectrum of chemical entities that are capable of engaging with genes implicated in lung cancer radiosensitivity [30]. It is worth highlighting that β‐Thy has been evidenced to interact with SOD1, thereby influencing the radiosensitivity of non‐small cell lung cancer. Previous research endeavors have corroborated that the downregulation of SOD1 can enhance the radiosensitivity of NSCLC by inhibiting the production of ROS and the pathways involved in DNA damage repair subsequent to radiotherapy [31, 32]. Nevertheless, the majority of previous research has primarily centered on the expression levels of SOD1 in lung cancer and its correlation with prognosis and therapeutic efficacy, with limited probing into the role of SOD1 in modulating radiosensitivity. Additionally, high‐performance liquid chromatography analyses have confirmed the presence of β‐Thy components in Maimendong Tang, further emphasizing the importance of β‐Thy in modulating radiosensitivity.

SOD1, a key cytoplasmic antioxidant enzyme, holds significant implications for NSCLC therapy. The nuclear enrichment of SOD1 is crucial for the proliferation of NSCLC cells, a fact corroborated by SOD1 inducible knockout studies in KRAS‐driven NSCLC mouse models, which significantly diminished the tumor burden in both in vivo and in vitro settings [33]. Clinical investigations have also highlighted the prognostic relevance of serum SOD1 levels, with elevated concentrations correlating with increased all‐cause mortality in patients with NSCLC. Moreover, epigenetic regulation studies revealed that SOD1 has a role in NSCLC progression and that its inhibition induces cell cycle arrest and apoptosis. Intriguingly, it has been demonstrated that the mechanisms underlying this phenomenon involve the miR‐409‐3p/SOD1/SETDB1 regulatory loop [3, 4]. Additionally, ceramide treatment has been found to trigger an increase in ROS, modulate the SOD1/SOD2 ratio, and hinder the proliferation of NSCLC cells, thereby suggesting potential therapeutic avenues [34, 35]. Therefore, SOD1 has emerged as a multifaceted player in NSCLC that influences tumorigenesis, patient prognosis, and treatment response. Our prior research has confirmed that SOD1, regulated by the CBR3‐AS1/miR‐409‐3p axis, can prevent the clearance of ROS induced by radiotherapy in mitochondria while enhancing the capacity for DNA damage repair, ultimately resulting in radiotherapy resistance in NSCLC cells [3]. Our results showed that SOD1 might be one of the targets of β‐Thy, a component of RBMD; thus, we proposed a hypothesis that β‐Thy might enhance radiosensitivity by inhibiting SOD1. The experimental results confirmed the capability of β‐Thy to influence SOD1 mRNA and protein expression, and the further single‐cell gel electrophoresis results demonstrated that β‐Thy enhances DNA damage following radiotherapy by reducing SOD1, impedes DNA repair, and promotes apoptosis. Consequently, we propose that the consumption of RBMD may mitigate the procarcinogenic effects of SOD1 on NSCLC cells via β‐Thy, promoting mitochondrial ROS accumulation induced by radiotherapy and reducing DNA damage repair capacity to enhance radiosensitivity. This study deepens our understanding of the mechanisms through which TCM remedies regulate NSCLC radiosensitivity and provides a theoretical basis for further exploration in clinical practice.

Our investigation still had certain limitations. First, due to the survival rate issues in the in vivo experiments, we had only three mice per group, which might have led to high data variability and errors. Second, in this study, we focused on SOD1, one of the target genes of β‐Thy, as our previous research had validated its involvement in the radioresistance of NSCLC [3]. As a classic traditional formula, RBMD showcases its ingenious composition through the multi‐dimensional synergistic effects of various herbs, which collectively regulate physiological functions and local tissue environments, thereby enhancing radiosensitivity. Therefore, we considered that other potential mechanisms could also contribute to the radioresistance of NSCLC, which might be targeted by different components in RBMD to enhance radiosensitivity. These additional mechanisms merit further exploration. Moreover, we acknowledge that the absence of specific batch analyses for β‐thy constitutes an inadequacy in characterizing analytical precision. Therefore, quality control measures will be implemented to ensure batch‐to‐batch consistency in RBMD formulations, which primarily focuses on verifying formula ratios and major bioactive constituents, particularly β‐thy. Finally, the clinical radiotherapy regimens are different from animal experiments. In our study, the doses of 8Gy/f and 4Gy/f that we adopted fall into the category of hypofractionated doses in clinical practice and require further exploration in preclinical models to bolster clinical validity.

To summarize, it is proposed that the combination of ingredients in RBMD may enhance the radiosensitivity of NSCLC cells. However, further research is required to explore the effects of TCM on NSCLC radiosensitivity and its prospects in clinical practice. The observed effect is likely mediated by the active ingredient β‐Thy, which binds SOD1 and reduces its expression. This process enhances DNA damage following radiotherapy, impedes DNA repair, and promotes apoptosis. Our findings confirm that β‐Thy has the capability to influence the expression of SOD1 at both mRNA and protein levels, thereby counteracting the oncogenic effects of SOD1 on the malignant characteristics of NSCLC cells. Therefore, we suggest that the consumption of RBMD, which is rich in β‐Thy, may mitigate the procarcinogenic effects of SOD1 on NSCLC cells, including cell proliferation, apoptosis, migration, invasion, and resistance to radiation therapy.

## Ethics Statement

All animal studies (including the mice euthanasia procedure) complied with the regulations and guidelines of Harbin Medical University Institutional Animal Care (KY2022‐57) and conducted according to The American Association for Laboratory Animal Science and the Institutional Animal Care and Use Committee Guidelines.

## Disclosure

No persons or third‐party services beyond the listed authors were involved in the research or in the preparation of this manuscript. All authors agree to be accountable for the content and conclusions of the article.

## Conflicts of Interest

The authors declare no conflicts of interest.

## Author Contributions

Shilong Liu and Xueying Pang performed the study, wrote the manuscript, and contributed equally to this work. Liqun Wang and Ning Zhan helped in most of the experiments. Ke Jin, Yang Bai, and Liqun Wang contributed to verifying the results and revising the manuscript. Shangjie Wu, Jiaxing Deng, and Lishuang Qi contributed to the statistical analyses. Deyou Jiang and Zhuying Li prepared the herbal formulas of RBMD.

## Funding

This work was supported by the Hai Yan Fund of The Third Affiliated Hospital of Harbin Medical University, Harbin, China (Grant JJZD2023‐01); the China Postdoctoral Science Foundation (Grant 2022MD713752); the Heilongjiang Postdoctoral Science Foundation (Grant LBH‐Z21081) to SLL; and the Outstanding Youth Foundation of Heilongjiang Province of China (Grant YQ2023H002) to LSQ.

## Supporting Information

Additional supporting information can be found online in the Supporting Information section.

## Supporting information


**Supporting Information 1** Figure S1: KEGG pathway enrichment plot of the five key RBMD target genes involved in NSCLC radiosensitivity.

**Supporting Information 2** Figure S2: The boxplots of SOD1 mRNA expression between cancer samples and normal controls in TCGA‐LUAD (a) and TCGA‐LUSC (b).

## Data Availability

The data that support the findings of this study are openly available in the Encyclopedia of Traditional Chinese Medicine (ETCM) database at http://www.tcmip.cn/ETCM/; TCGA at https://portal.gdc.cancer.gov/; CRISPR‐Cas9 screening dataset in the DepMap portal at https://depmap.org/portal/download/; dbCRSR at http://www.xialab.info:8080/dbCRSR/index.jsp; CB‐Dock2 server at https://cadd.labshare.cn/cb-dock2/php/index.php.

## References

[1] Siegel R. L. , Miller K. D. , and Jemal A. , Cancer Statistics, 2020, CA: A Cancer Journal for Clinicians. (2020) 70, no. 1, 7–30, 10.3322/caac.21590. 31912902

[2] Duma N. , Santana-Davila R. , and Molina J. R. , Non–Small Cell Lung Cancer: Epidemiology, Screening, Diagnosis, and Treatment, Mayo Clinic Proceedings. (2019) 94, no. 8, 1623–1640, 10.1016/j.mayocp.2019.01.013, 2-s2.0-85069823964. 31378236

[3] Liu S. , Zhan N. , and Gao C. , et al.Long Noncoding RNA CBR3-AS1 Mediates Tumorigenesis and Radiosensitivity of Non-Small Cell Lung Cancer Through Redox and DNA Repair by CBR3-AS1 /miR-409-3p/SOD1 Axis, Cancer Letters. (2022) 526, 1–11, 10.1016/j.canlet.2021.11.009. 34801596

[4] Liu S. , Li B. , and Xu J. , et al.SOD1 Promotes Cell Proliferation and Metastasis in Non-Small Cell Lung Cancer via an miR-409-3p/SOD1/SETDB1 Epigenetic Regulatory Feedforward Loop, Frontiers in Cell and Developmental Biology. (2020) 8, 10.3389/fcell.2020.00213, 213. 32391354 PMC7190798

[5] Zhang Y. , Li X. , and Shi Y. , et al.ETCM v2.0: An Update With Comprehensive Resource and Rich Annotations for Traditional Chinese Medicine, Acta Pharmaceutica Sinica B. (2023) 13, no. 6, 2559–2571, 10.1016/j.apsb.2023.03.012. 37425046 PMC10326295

[6] Tan L. , Wang W. , and He G. , et al.Resveratrol Inhibits Ovarian Tumor Growth in an in Vivo Mouse Model, Cancer. (2016) 122, no. 5, 722–729, 10.1002/cncr.29793, 2-s2.0-84958832382. 26619367

[7] Fang Y. , DeMarco V. G. , and Nicholl M. B. , Resveratrol Enhances Radiation Sensitivity in Prostate Cancer by Inhibiting Cell Proliferation and Promoting Cell Senescence and Apoptosis, Cancer Science. (2012) 103, no. 6, 1090–1098, 10.1111/j.1349-7006.2012.02272.x, 2-s2.0-84861682542. 22417066 PMC7685088

[8] Xue L. , Encyclopedia of Chinese Herbs, 2019, Science and Technology Press, Heilongjiang.

[9] Jiang D. , Dragon River School of Medicine Series - Cold Land Health Care, 2018, Science Press, Beijing.

[10] Feng Y. , Guo R. , Qu Y. , and Qu F. , Effects of *Agaricus blazei* Polysaccharide Combined With Selenium on the Metabolic Effects of *Agaricus blazei* in Pancreatic Cancer Malignant Mice, Journal of Sichuan North Medical College. (2020) 35, no. 3, 5.

[11] Wang S. , Yang Y. , and Xu L. , et al.Materia Medica of Pingbeimu and Beimu-Like Herbs, Journal of Liaoning University of Traditional Chinese Medicine. (2021) no. 9.

[12] Xiao Y. , Li P. , and Shu Q. , Inhibition of Human Lung Cancer A549 Cells by Aqueous Extracts of Southern Red Bean Curd Combined With Paclitaxel and Effects on Drug Resistance Genes, Chinese Journal of Traditional Chinese Medicine. (2020) 35, no. 11, 4.

[13] Yueheng Zhao S. C. , Shi R. , and Zhang H. , Study on the Mechanism of Action of Maimendong Tang on Autophagy and Lung Water Clearance of Type II Alveolar Epithelial Cells in Mice With Interstitial Pneumonia, Journal of Zhejiang University of Traditional Chinese Medicine. (2021) 45, no. 2, 8.

[14] Xu H. Y. , Zhang Y. Q. , and Liu Z. M. , et al.ETCM: An Encyclopaedia of Traditional Chinese Medicine, Nucleic Acids Research. (2019) 47, no. D1, D976–D982, 10.1093/nar/gky987, 2-s2.0-85059796319. 30365030 PMC6323948

[15] Jia D. , Li S. , Li D. , Xue H. , Yang D. , and Liu Y. , Mining TCGA Database for Genes of Prognostic Value in Glioblastoma Microenvironment, Aging. (2018) 10, no. 4, 592–605, 10.18632/aging.101415. 29676997 PMC5940130

[16] Korostin D. , Kulemin N. , Naumov V. , Belova V. , Kwon D. , and Gorbachev A. , Comparative Analysis of Novel MGISEQ-2000 Sequencing Platform Vs Illumina HiSeq 2500 for Whole-Genome Sequencing, PLOS ONE. (2020) 15, no. 3, 10.1371/journal.pone.0230301, e0230301. 32176719 PMC7075590

[17] Yoshihara K. , Shahmoradgoli M. , and Martínez E. , et al.Inferring Tumour Purity and Stromal and Immune Cell Admixture From Expression Data, Nature Communications. (2013) 4, no. 1, 10.1038/ncomms3612, 2-s2.0-84885673911, 2612. PMC3826632 24113773

[18] Zheng H. , Song K. , and Fu Y. , et al.An Absolute Human Stemness Index Associated With Oncogenic Dedifferentiation, Briefings in Bioinformatics. (2021) 22, no. 2, 2151–2160, 10.1093/bib/bbz174. 32119069

[19] Whitfield M. L. , George L. K. , Grant G. D. , and Perou C. M. , Common Markers of Proliferation, Nature Reviews Cancer. (2006) 6, no. 2, 99–106, 10.1038/nrc1802, 2-s2.0-33644834789. 16491069

[20] Eustace A. , Mani N. , and Span P. N. , et al.A 26-Gene Hypoxia Signature Predicts Benefit From Hypoxia-Modifying Therapy in Laryngeal Cancer but Not Bladder Cancer, Clinical Cancer Research. (2013) 19, no. 17, 4879–4888, 10.1158/1078-0432.CCR-13-0542, 2-s2.0-84883470111. 23820108 PMC3797516

[21] Su W. , Hong T. , Feng B. , Yang Z. , and Lei G. , A Unique Regulated Cell Death-Related Classification Regarding Prognosis and Immune Landscapes in Non-Small Cell Lung Cancer, Frontiers in Immunology. (2023) 14, 10.3389/fimmu.2023.1075848, 1075848. 36817452 PMC9936314

[22] Hänzelmann S. , Castelo R. , and Guinney J. , GSVA: Gene Set Variation Analysis for Microarray and RNA-Seq Data, BMC Bioinformatics. (2013) 14, no. 1, 10.1186/1471-2105-14-7, 2-s2.0-84872202078, 7. 23323831 PMC3618321

[23] Izeradjene K. , Revillard J.-P. , and Genestier L. , Inhibition of Thymidine Synthesis by Folate Analogues Induces a Fas–Fas Ligand-Independent Deletion of Superantigen-Reactive Peripheral T Cells, International Immunology. (2001) 13, no. 1, 85–93, 10.1093/intimm/13.1.85, 2-s2.0-0035176312. 11133837

[24] Zhang H. , Lin Y. , Zhuang M. , Zhu L. , Dai Y. , and Lin M. , Screening and Identification of CNIH4 Gene Associated With Cell Proliferation in Gastric Cancer Based on a Large-Scale CRISPR-Cas9 Screening Database DepMap, Gene. (2023) 850, 10.1016/j.gene.2022.146961, 146961. 36220450

[25] Wen P. , Xia J. , and Cao X. , et al.DbCRSR: A Manually Curated Database for Regulation of Cancer Radiosensitivity, Database. (2018) 2018, 10.1093/database/bay049, 2-s2.0-85054778157. PMC6007213 29860480

[26] Trott O. and Olson A. J. , AutoDock Vina: Improving the Speed and Accuracy of Docking With a New Scoring Function, Efficient Optimization, and Multithreading, Journal of Computational Chemistry. (2010) 31, no. 2, 455–461, 10.1002/jcc.21334, 2-s2.0-76149120388. 19499576 PMC3041641

[27] Wu S. , Li Z. , Li H. , and Liao K. , Dihydroartemisinin Reduces Irradiation-Induced Mitophagy and Radioresistance in Lung Cancer A549 Cells via CIRBP Inhibition, Life. (2022) 12, no. 8, 10.3390/life12081129, 1129. 36013308 PMC9410454

[28] Jin B. , Kong W. , and Zhao X. , et al.Substrate Stiffness Affects the Morphology, Proliferation, and Radiosensitivity of Cervical Squamous Carcinoma Cells, Tissue and Cell. (2022) 74, 10.1016/j.tice.2021.101681, 101681. 34837739

[29] Bader S. , Wilmers J. , Pelzer M. , Jendrossek V. , and Rudner J. , Activation of Anti-Oxidant Keap1/Nrf2 Pathway Modulates Efficacy of Dihydroartemisinin-Based Monotherapy and Combinatory Therapy With Ionizing Radiation, Free Radical Biology and Medicine. (2021) 168, 44–54, 10.1016/j.freeradbiomed.2021.03.024. 33775773

[30] Ma Y. H. , Liu Y. , and Li T. , et al.An Experimental Study on the Visual Identification of Fritillaria Ussuriensis Based on LAMP and Nucleic Acid Colloidal Gold Technique, Analytical Biochemistry. (2024) 687, 10.1016/j.ab.2023.115430, 115430. 38147947

[31] Liu T. , Li K. , and Zhang Z. , et al.Tetrandrine Inhibits Cancer Stem Cell Characteristics and Epithelial to Mesenchymal Transition in Triple-Negative Breast Cancer via SOD1/ROS Signaling Pathway, The American Journal of Chinese Medicine. (2023) 51, no. 2, 425–444, 10.1142/S0192415X23500222. 36692485

[32] Dimayuga F. O. , Wang C. , Clark J. M. , Dimayuga E. R. , Dimayuga V. M. , and Bruce-Keller A. J. , SOD1 Overexpression Alters ROS Production and Reduces Neurotoxic Inflammatory Signaling in Microglial Cells, Journal of Neuroimmunology. (2007) 182, no. 1-2, 89–99, 10.1016/j.jneuroim.2006.10.003, 2-s2.0-33845890520. 17097745 PMC1797892

[33] Wang X. , Zhang H. , and Sapio R. , et al.SOD1 Regulates Ribosome Biogenesis in KRAS Mutant Non-Small Cell Lung Cancer, Nature Communications. (2021) 12, no. 1, 10.1038/s41467-021-22480-x, 2259. PMC8050259 33859191

[34] Chang Y. C. , Fong Y. , and Tsai E. M. , et al.Exogenous C_8_-Ceramide Induces Apoptosis by Overproduction of ROS and the Switch of Superoxide Dismutases SOD1 to SOD2 in Human Lung Cancer Cells, International Journal of Molecular Sciences. (2018) 19, no. 10, 10.3390/ijms19103010, 2-s2.0-85054371316. PMC6213533 30279365

[35] Glasauer A. , Sena L. A. , Diebold L. P. , Mazar A. P. , and Chandel N. S. , Targeting SOD1 Reduces Experimental Non–small-Cell Lung Cancer, The Journal of clinical investigation. (2014) 124, no. 1, 117–128, 10.1172/JCI71714, 2-s2.0-84892948219. 24292713 PMC3871252

