# Supplementary figures and images for: Rongbei Maimendong Decoction Promotes Radiosensitivity of Non‐Small Cell Lung Cancer Cells by Inhibiting SOD1 Expression
Source: Mediators Inflamm. 2025 Dec 8;2025:3930562. doi: 10.1155/mi/3930562 (PMC12767424; doi:10.1155/mi/3930562)

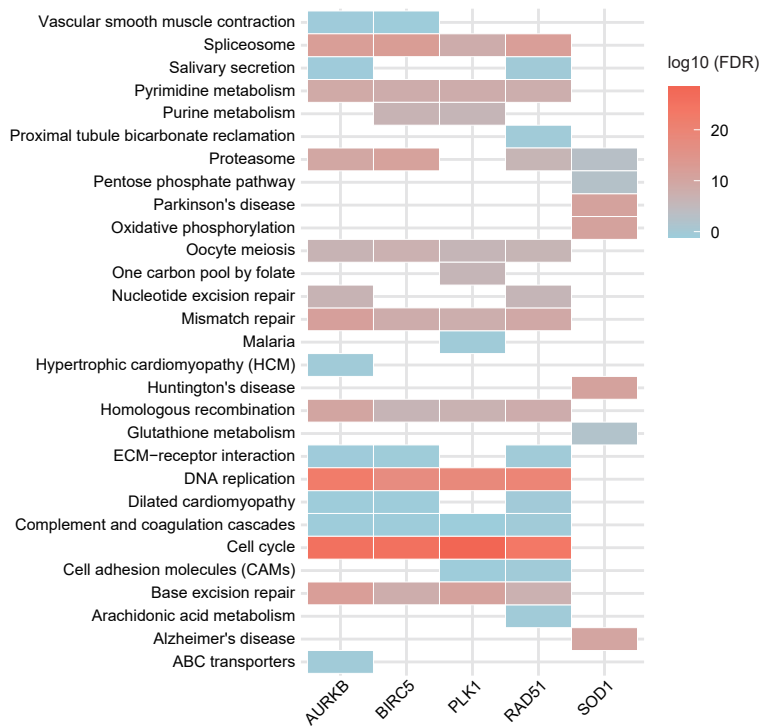

Supplement: Supplementary file 1 — Supporting Information 1 Figure S1: KEGG pathway enrichment plot of the five key RBMD target genes involved in NSCLC radiosensitivity. [file MI-2025-3930562-s002.pdf]

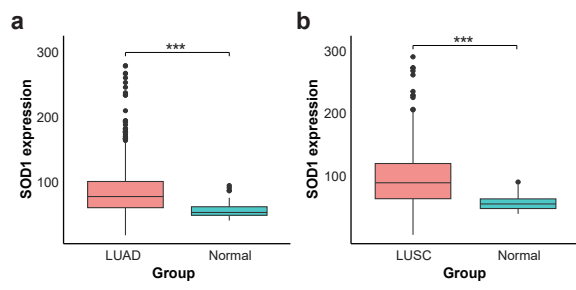

Supplement: Supplementary file 2 — Supporting Information 2 Figure S2: The boxplots of SOD1 mRNA expression between cancer samples and normal controls in TCGA‐LUAD (a) and TCGA‐LUSC (b). [file MI-2025-3930562-s001.pdf]
